# Practical but Inaccurate? A-Mode Ultrasound and Bioelectrical Impedance Underestimate Body Fat Percentage Compared to Dual-Energy X-ray Absorptiometry in Male College Students

**DOI:** 10.3390/jfmk9030113

**Published:** 2024-06-28

**Authors:** Markus Olinto, Victor César Lins, Gabriel Rocha, Marco Aurélio Dourado, Maurilio Dutra

**Affiliations:** 1 Faculty of Physical Education, University of Brasília, Brasilia 70910-900, Brazil; markusolinto@gmail.com (M.O.); gabricolico@gmail.com (G.R.); 2 Exercise and Health Research Group, Campus Estrutural, Federal Institute of Education, Science and Technology of Brasilia, Brasilia 71200-020, Brazil; victorcesardiaslins@gmail.com (V.C.L.); douradopersonal@gmail.com (M.A.D.)

**Keywords:** body fat, bioelectrical impedance, ultrasound, dual-energy X-ray absorptiometry

## Abstract

Bioelectrical impedance (BIA) and ultrasound (US) have become popular for estimating body fat percentage (BF%) due to their low cost and clinical convenience. However, the agreement of these devices with the gold-standard method still requires investigation. The aim was to analyze the agreement between a gold-standard %BF assessment method with BIA and US devices. Twenty-three men (aged 30.1 ± 7.7 years, weighing 82.5 ± 14.9 kg, 1.77 ± 0.05 m tall) underwent dual-energy X-ray absorptiometry (DXA), BIA (tetrapolar) and US (three-site method) %BF assessments. Pearson and concordance correlations were analyzed. A *T*-test was used to compare the means of the methods, and Bland–Altman plots analyzed agreement and proportional bias. Alpha was set at <0.05. The Pearson coefficients of BIA and US with DXA were high (BIA = 0.94; US = 0.89; both *p* < 0.001). The concordance coefficient was high for BIA (0.80) and moderate for US (0.49). The BF% measured by BIA (24.5 ± 7.5) and US (19.4 ± 7.0) was on average 4.4% and 9.6% lower than DXA (29.0 + 8.5%), respectively (*p* < 0.001). Lower and upper agreement limits between DXA and BIA were −1.45 and 10.31, while between DXA and US, they were 2.01 and 17.14, respectively. There was a tendency of both BIA (*p* = 0.09) and US (*p* = 0.057) to present proportional bias and underestimate BF%. Despite the correlation, the mean differences between the methods were significant, and the agreement limits were very wide. This indicates that BIA and US, as measured in this study, have limited potential to accurately measure %BF compared to DXA, especially in individuals with higher body fat.

## 1. Introduction

Body composition assessment and research date back to the 1940s and 1950s [1,2]. They became more relevant and advanced together with the increase in obesity and related chronic diseases in the mid-1970s, as well as with the identification of sarcopenia as a relevant health concern in the late 1980s [3]. Since then, a wide range of techniques have been developed trying to accurately assess body composition (i.e., body fat and muscle mass) in children/teenagers, adults and the elderly. Of note, valid and accurate body composition assessment is essential for diagnostic and clinical purposes in health and disease [4].

There are some powerful and accurate reference methods to distinguish body mass components, such as underwater weighing, air replacement plethysmography, neutron activation analysis, computed tomography and dual-energy X-ray densitometry (DXA) [5]. Among these, DXA is widely used as a gold-standard reference method to assess the accuracy and agreement of other methods [6,7,8,9,10].

In clinical settings, such as during nutritional evaluation and ambulatory visits, as well as in sports, fitness or wellness contexts, a wide range of indirect methods have been used to assess body fat percentage due to the fact that they are low cost, less time-consuming, transportable and have no side effects [5]. The most used indirect methods include calculation by analytical formulas from simple anthropometric traits and caliper testing. Of note, bioelectrical impedance (BIA) and ultrasound (US) scanning have gained attention in clinical and research studies [5,8].

BIA devices are generally simple to use, inexpensive and avoid radiation exposure. This method is based on the electrical properties of the body and determines the resistance resulting from an electrical current passing through the body [11]. The subject’s weight, height and age are considered to estimate total body water. Then, specific equations are applied to determine the body fat percentage (BF%) [6]. Several BIA devices are commercially available, and they have been applied to assess body fat in obese people, young men and women and to analyze the risk of osteoporosis development [11].

US techniques have also become common in the last decade. These devices identify adipose tissue using ultrasound waves that travel into body tissues. This method is non-invasive and non-traumatizing to the subject [12]. So, some studies have been trying to assess US accuracy to measure body fat in different populations, such as male college students [13] and athletes [14]. Thus, BIA and US have been also proposed as possible alternatives to assess and monitor BF% in the general population.

However, both BIA and US are not free from potential bias. As they are indirect methods, several factors may influence the result. Height, sex, age, ethnicity, total body water, body sites of US measurement (protocol), nutritional status and physical activity are among possible confounding variables to the measurements [5,12]. Furthermore, several equipment models are available, which makes it difficult to establish standard protocols. In this sense, the validation and agreement of these procedures with gold-standard methods are still required.

The present study aimed to assess the agreement of measurement of BF% obtained by a tetrapolar bioimpedance analyzer and an A-mode US scanner using a three-site protocol compared to DXA in a group of male college students.

## 2. Materials and Methods

### 2.1. Participants and Study Design

This is a cross-sectional study. A convenience sample of healthy male college students was recruited to participate. The inclusion criterion was enrollment in any undergraduate or graduate course at the university. Data collection occurred in the Image Laboratory of the Faculty of Physical Education. All men who volunteered to participate and gave informed consent were included in the study. Twenty-three subjects completed all the analysis. 

### 2.2. BF% Assessment

All subjects underwent BF% assessment in the morning, between 9 h and 11 h, in the following order: BIA, US and DXA. Volunteers were dressed in light clothes and were instructed to remove all jewelry and metals prior to examinations. A tetrapolar BIA device was used in this study (OMROM HBF 514-C^®^, Omron Healthcare Co., Ltd., Kyoto, Japan) to assess body weight and BF%. The BIA sends a weak electric current (50 kHz in the present device) through the body, and the resulting voltage is used to calculate bioelectrical impedance, which is divided into resistance and reactance. These measurements can be used to estimate total body water, fat-free mass and fat mass [11]. It is important to note that the exact equation used by this specific BIA device is proprietary and not publicly known. The height of the participants was also evaluated using a wall stadiometer (Sanny®, São Paulo, Brazil) and body mass index (BMI) was derived from Quetelet’s formula (weight/height^2^).

An A-mode, 2.5 MHz, portable US device (BodyMetrix BX2000^®^ system, Intela Metrix, Concord, CA, USA) was used. The US emits high-frequency sound waves to penetrate body tissues. Differentiation of body tissue interfaces is determined based on the thickness of tissue and the length of time it takes for the ultrasonic waves to pass through and reflect back into the transducer [8]. Measurements were performed according to the manufacturer’s instructions. A thin layer of US gel was applied to the probe and then placed perpendicular to the point of skin contact at each site. The Bodyview^®^ software (version BodyView ProFit 3.0.9.22073-N, IntelaMetrix, Concord, CA, USA) was used to analyze images and to measure the thickness of adipose tissue at each site. BF% was derived from calculations using the thickness of the adipose layer of the chest, abdomen and thigh, analogous with the skinfold method and adapted from the Jackson–Pollock three-site protocol for men [15]. All evaluations were performed by the same trained, certified technician.

DXA-derived BF% was measured using a Lunar densitometer, model DPX (General Eletric-GE, Rommelsdorf, Germany). The equipment was calibrated daily and weekly according to the manufacturer’s instructions. Participants were instructed to lie quietly in a supine position with their arms at their sides on the scanning bed. Scanning of the entire body was performed.

### 2.3. Statistical Analysis

The normality of the data was assessed using the Shapiro–Wilk test. All BF% measurements presented parametric distribution. Correlations between BMI-, BIA-, US- and DXA-derived BF% were analyzed with the Pearson coefficient and the concordance correlation coefficient (simple agreement analysis). One sample *T*-test was used to compare the mean differences between BF% derived from DXA and the other methods (DXA—BIA and DXA—US). The Bland–Altman plots were used to analyze agreement between DXA and BIA, as well as between DXA and US. Limits of agreement were set at a confidence and agreement level of 95%. A linear regression analysis was used to investigate proportion bias related to BIA and US when compared to DXA. All analysis was conducted using the software Jamovi for Windows, version 2.3.28.

## 3. Results

Descriptive characteristics of the subjects, as well as the measured BF% from DXA, BIA and US, are presented in Table 1. The mean BMI showed that the sample was slightly overweight.

The BF%s derived by BIA and US were highly correlated with DXA when considering the Pearson correlation coefficient (r = 0.94 and 0.89 for BIA and US, respectively, both *p* < 0.001). When considering the concordance correlation derived from simple agreement analysis, the coefficients were 0.80 for BIA and 0.49 for US. BMI was also highly correlated with all BF% methods (r = 0.85; 0.93; and 0.84 for DXA, BIA and US, respectively. All *p* < 0.001).

### 3.1. Agreement between DXA and BIA

**Figure 1:**
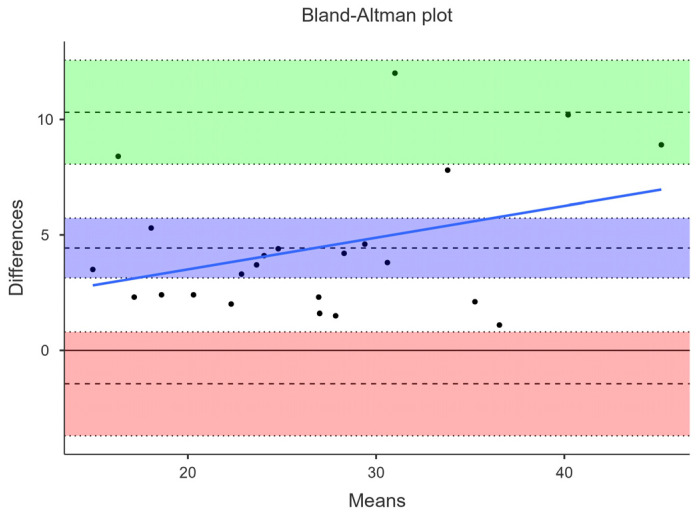
Bland–Altman plot. Comparison between BF% derived by DXA and BIA. Green and salmon color areas represent the estimate of the upper and lower limit of agreement (dashed line in the center is the estimate) with lower and upper confidence intervals (dotted lines at the bottom and top of the green and salmon area). Lilac color represents the mean difference between methods (dashed line) with lower and upper confidence intervals (dotted lines at the bottom and top of the lilac color area).

The concordance correlation coefficient was high for BIA (0.80). Yet, BF% measured by BIA (24.5 ± 7.5) was on average 4.4% lower than DXA (29.0 ± 8.5). This mean difference was statistically significant (*p* < 0.001). The Bland–Altman plot showed very large limits of agreement between DXA and BIA. The lower and upper agreement limits were −1.45 (95% CI −3.70–0.80) and 10.31 (95% CI 8.06–12.56). There was a tendency of BIA to underestimate BF%, especially among those with higher BF%, as shown by the blue proportional bias line in Figure 1. Linear regression confirmed this tendency of proportional bias of BIA (*p* = 0.09) to underestimate BF% when compared to DXA.

### 3.2. Agreement between DXA and US

**Figure 2:**
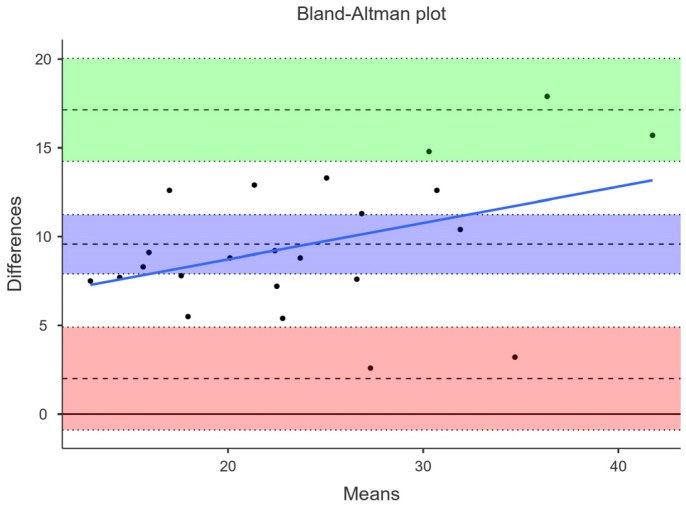
Bland–Altman plot. Comparison between BF% derived by DXA and US. Green and salmon color areas represent the estimate of the upper and lower limit of agreement (dashed line in the center is the estimate) with lower and upper confidence intervals (dotted lines at the bottom and top of the green and salmon area). Lilac color represents the mean difference between methods (dashed line) with lower and upper confidence intervals (dotted lines at the bottom and top of the lilac color area).

The concordance correlation coefficient was moderate for US (0.49). BF% measured by US (19.4 ± 7.0) was on average 9.6% lower than DXA (29.0 + 8.5%). This mean difference was statistically significant (*p* < 0.001). The Bland–Altman plot showed very large limits of agreement between DXA and US. The lower and upper agreement limits were 2.01 (95% CI −0.89–4.90) and 17.14 (95% CI 14.25–20.04). The tendency to underestimate BF%, especially among those with higher BF%, was more pronounced in US, as shown by the proportional bias line (blue) in Figure 2. Linear regression confirmed this proportional bias tendency of US (*p* = 0.057) to underestimate BF% when compared to DXA.

## 4. Discussion

This study aimed to investigate the agreement of measurement of BF% obtained by BIA and US compared to DXA in a group of healthy male college students. The main result was that, although the methods showed a high correlation, both BIA and US significantly underestimated BF% compared to the gold-standard reference (DXA). This underestimation was more pronounced among those with high BF%.

BIA has been widely used in research settings to assess the body composition of adults [4], older adults [16], people with chronic disease [7] and male and female college students [17]. There are several BIA devices that are commercially available, and some were tested for their validity and agreement with gold-standard methods.

Although we did not find a single study testing the BIA model that was used in the present study, Pribyl, Smith and Grimes [17] analyzed the accuracy of a very similar model (Omrom HBF-500) within a sample of male and female college students (±25.8 years). They found that BIA significantly overestimated BF% in males by approximately 1.5% with tighter agreement limits, while the present study found an underestimation of BF% by BIA with large agreement limits. However, they compared BIA with air displacement plethysmography (BOD POD) as the criterion, while this study used DXA as the reference. So, further comparison is limited. Furthermore, another study with young men compared BIA devices with BOD POD as the reference and found no significant differences regarding BF% [18].

Rockamann et al. [10] compared four different BIA devices with DXA in a sample of male and female college students (±19.8 years). All devices were hand-held. In the present study, a tetrapolar hand and foot device was analyzed, limiting comparison. Despite that, they found that two of the four devices underestimate BF% by −3.6 and −5.8% even with a moderate correlation coefficient (around 0.64), which is similar to the result of the present study (we found a mean difference of −4.5% with a high correlation).

Just like BIA, US has been used in research and clinical settings for its low cost, ease of use and transportation [9]. Similar to BIA, a variety of US devices are available, and some have been considered useful to assess subcutaneous fat tissue [19] and to predict body fat [14]. Of note, some previous studies investigated the validity of the device that we used in the present study (BodyMetrix B2000). This device measures the thickness of the subcutaneous fat layer on specific body sites, which are then used to estimate body fat. This is analogous to the skinfold protocols proposed by Jackson and Pollock [15]. A comparison of caliper and US regarding the thickness of subcutaneous fat was performed previously but was not within the scope of the present work [19].

Johnson and colleagues [12] found that the BodyMetrix B2000 (BMB2000) failed to agree with DXA when measuring the BF% of male college students (±23.0 years) with a significant underestimation of −4.4%, even though there was a strong correlation (r = 0.84). This result is similar to what was found in the present study, but the underestimation here was even higher, reaching −9.6%, with a moderate concordance correlation of 0.49. Differences could be related to the fact that the present study used a three-site protocol, whereas Johnson et al. used a seven-site protocol.

Indeed, the agreement of different BMB2000 protocols may vary. Baranauskas and colleagues [8] observed significant differences in the BF% of male and female college students (±22.8 years) between seven-site and three-site Jackson and Pollock protocols, showing that the three-site BF% was significantly lower than the seven-site. In addition, both three-site (−5.1%) and seven-site (−3.9%) protocols significantly underestimated BF% when compared to DXA. Similar to the Baranauskas study, Elsey and colleagues [20] also found the three-site protocol to underestimate BF% compared to seven-site in a sample of female athletes.

One study performed a thorough investigation about various BMB2000 protocols in male college students (±20.0 years) and compared the results to DXA [13]. The authors observed that the three-site protocol, as used in the present study, showed a strong Pearson correlation with DXA (r = 0.87). In the present study, the Pearson correlation was very similar (r = 0.89), but the concordance correlation coefficient, which is more adequate to test agreement between methods, was moderate in the present findings (0.49). Moreover, Kang et al. [13] observed an underestimation of about −7.0% compared to DXA-measured BF%, and this was the worst of all nine protocols they evaluated.

Strengths and limitations are recognized in the present study. Firstly, we assessed a small convenience sample, which may be statistically underpowered. However, a gold-standard method that is difficult to access was used as the reference. Secondly, only one US protocol was analyzed. However, we chose the three-site protocol due to its alleged accessibility and suitability in clinical settings. Of note, Jackson and Pollock’s equation has been validated in Brazilian males [21] and is extensively used in body composition research in Brazil. Thirdly, only adult men attending college were included, which makes it impossible to generalize the results to other groups. Noteworthy, this study analyzed a BIA device that was not analyzed before (to the best of our knowledge). Finally, there were no specific instructions regarding hydration or fasting before the measurements, as the sample was acquired through convenience. This may introduce bias to comparison with other studies. Yet, it is important to mention that this approach mirrors a commercial setting, where participants typically arrive without prior instructions concerning exercise and food and water consumption.

## 5. Conclusions

In summary, BIA and US have been considered effective alternatives to the expensive and technical DXA technique [12]. However, these methods also require practical device operation skills and proper training before the examination, especially US, because of details like proper probe positioning [22]. In the present study, both devices showed to be inaccurate to estimate BF% compared to DXA, even though they presented moderate to high correlation. This inaccuracy was higher in individuals with higher BF measured by DXA. Underestimation and proportional bias were more pronounced in the US method.

These results are consistent with the previous literature. Therefore, the risk of (mis)interpretation and bias is clear and may potentially impact nutritional and physical activity planning in clinical settings [23]. BF% underestimation may supposedly lead to accommodation and lack of engagement in nutritional and physical activity programs among healthy, overweight and obese people. So, it is possible that simple and inexpensive methods of BF% assessment may negatively interfere in BF monitoring [10].

Future studies should continue to investigate the validity and agreement between new body composition devices and reference methods with diverse and larger samples, as body composition monitoring is essential in health and many disease contexts.

## Figures and Tables

**Table 1:** Descriptive characteristics of the subjects (mean ± SD), n = 23.

**Variable**	**Mean**	SD *
Age (years)	30.1	7.7
Weight (kg)	82.5	14.9
Height (m)	1.77	0.05
BMI (kg/m^2^)	26.3	4.37
DXA BF%	29.0	8.5
BIA BF%	24.5	7.5
US BF%	19.4	7.0

* SD: standard deviation. BMI: body mass index. BF%: body fat percentage. DXA: dual-energy X-ray absorptiometry. BIA: bioelectrical impedance. US: ultrasound.

## Data Availability

Data are contained within this article.
